# FASN-dependent de novo lipogenesis is required for brain development

**DOI:** 10.1073/pnas.2112040119

**Published:** 2022-01-07

**Authors:** Daniel Gonzalez-Bohorquez, Isabel M. Gallego López, Baptiste N. Jaeger, Sibylle Pfammatter, Megan Bowers, Clay F. Semenkovich, Sebastian Jessberger

**Affiliations:** ^a^Laboratory of Neural Plasticity, Faculties of Medicine and Science, Brain Research Institute, University of Zurich 8057 Zurich, Switzerland;; ^b^Functional Genomics Center Zurich, University of Zurich, Eidgenössiche Technische Hochschule Zurich, Zurich 8057, Switzerland;; ^c^Division of Endocrinology, Metabolism & Lipid Research, Washington University School of Medicine, St. Louis, MO 63108

**Keywords:** neurogenesis, neural stem cell, lipogenesis, polarity

## Abstract

Fate and behavior of neural progenitor cells are tightly regulated during mammalian brain development. Metabolic pathways, such as glycolysis and oxidative phosphorylation, that are required for supplying energy and providing molecular building blocks to generate cells govern progenitor function. However, the role of de novo lipogenesis, which is the conversion of glucose into fatty acids through the multienzyme protein fatty acid synthase (FASN), for brain development remains unknown. Using Emx1Cre-mediated, tissue-specific deletion of *Fasn* in the mouse embryonic telencephalon, we show that loss of FASN causes severe microcephaly, largely due to altered polarity of apical, radial glia progenitors and reduced progenitor proliferation. Furthermore, genetic deletion and pharmacological inhibition of FASN in human embryonic stem cell–derived forebrain organoids identifies a conserved role of FASN-dependent lipogenesis for radial glia cell polarity in human brain organoids. Thus, our data establish a role of de novo lipogenesis for mouse and human brain development and identify a link between progenitor-cell polarity and lipid metabolism.

## Significance

Regulation of cellular metabolism in proliferating progenitor cells and their neuronal progeny is critical for brain development and function. Here, we identify a pivotal role of fatty acid synthase (FASN)-dependent de novo lipogenesis for mouse and human brain development, as genetic deletion of FASN leads to microcephaly in the developing mouse cortex and cortical malformations in human embryonic stem cell–derived forebrain organoids. Mechanistically, we show that FASN is required for proper polarity of apical progenitor cells. The dual approach applied here, using mouse genetics and human forebrain organoids, establishes a role of FASN-dependent lipogenesis for mouse and human brain development and identifies a link between progenitor-cell polarity and lipid metabolism.

The formation of the mammalian brain depends on complex molecular mechanisms controlling the fate of neural progenitors, the number of neural cells generated, the correct differentiation and positioning of neural progeny, and the proper connectivity of individual neural cells and subregions (1–4). Prior research focused especially on the development of the mammalian cortex, given its role in higher cognitive functions and its substantial changes in size and connectivity that occurred with evolution (5–7).

In the developing cortex, there are two major classes of neural progenitor cells (NPCs): apical progenitors (APs) that divide in the ventricular zone (VZ) lining the ventricles and basal progenitor cells (BPs) that are derived from APs and divide within the subventricular zone (SVZ) to generate neuronal progeny (8–12). Early during embryonic development, APs (also referred to as radial glia cells given their long apical processes that span from the ventricle to the pial surface) initially expand via symmetric duplicating cell divisions before they enter asymmetric divisions, generating one self-renewed AP and either directly a neuronal daughter cell or an intermediate BP (10, 13–15). Based on genetics, single-cell RNA-sequencing (scRNA-seq), in vivo perturbations, and a number of transcriptional programs have been identified that regulate the distinct developmental steps orchestrating the generation of the correct number and layer-specific type of neurons generated before the onset of gliogenesis that occurs after most neuronal cells that are populating the cortex are born (16–18).

Recent data indicated that cell type– and state-dependent shifts in cellular metabolism, required for energy supply and for providing molecular building blocks for proliferating and newborn cells, are critically involved in the formation of the mammalian brain (19, 20). For example, it has been shown that APs are highly glycolytic while proliferative BPs metabolically shift toward oxidative phosphorylation (OXPHOS) (21–23). Furthermore, the breakdown of fatty acids (β-oxidation) into Acetyl-CoA (Acetyl coenzyme A) to fuel the tricarboxylic acid (TCA) cycle is necessary for normal brain development (24, 25). Indeed, a number of genetic diseases, such as certain forms of microcephaly, have been linked to alterations in glycolysis, β-oxidation, and glutaminolysis (26). However, a role for de novo lipogenesis, a major metabolic process in which the multiprotein enzyme fatty acid synthase (FASN) converts glucose into palmitate, the building block for more complex fatty acids, in the context of embryonic brain development remains unexplored (19, 20). Interestingly, previous studies showed that FASN is required for progenitor-cell divisions in adult neurogenic niches such as the hippocampus and the adult SVZ (27, 28). A recessive point variant in the human *FASN* gene, causing an enzymatic gain of function, has been associated with intellectual disability and affects NPCs (29, 30). In addition, de novo mutations in FASN have been associated with developmental epileptic encephalopathy (31), further indicating relevance of FASN-dependent lipid metabolism for human brain development and disease.

To probe for a potential role of FASN-dependent lipogenesis in mammalian brain development, we conditionally deleted Fasn in the developing mouse cortex and in human embryonic stem cell (hESC)-derived forebrain organoids. We show that genetic deletion and pharmacological inhibition of FASN causes a microcephalic phenotype due to altered polarity of APs, subsequent disruption of VZ cellular architecture, and reduced progenitor proliferation. Thus, our findings identify de novo lipogenesis as a key metabolic process ensuring proper development of the mouse and human brain.

## Results

### Genetic Deletion of Fasn Causes Microcephaly.

**Fig. 1.**
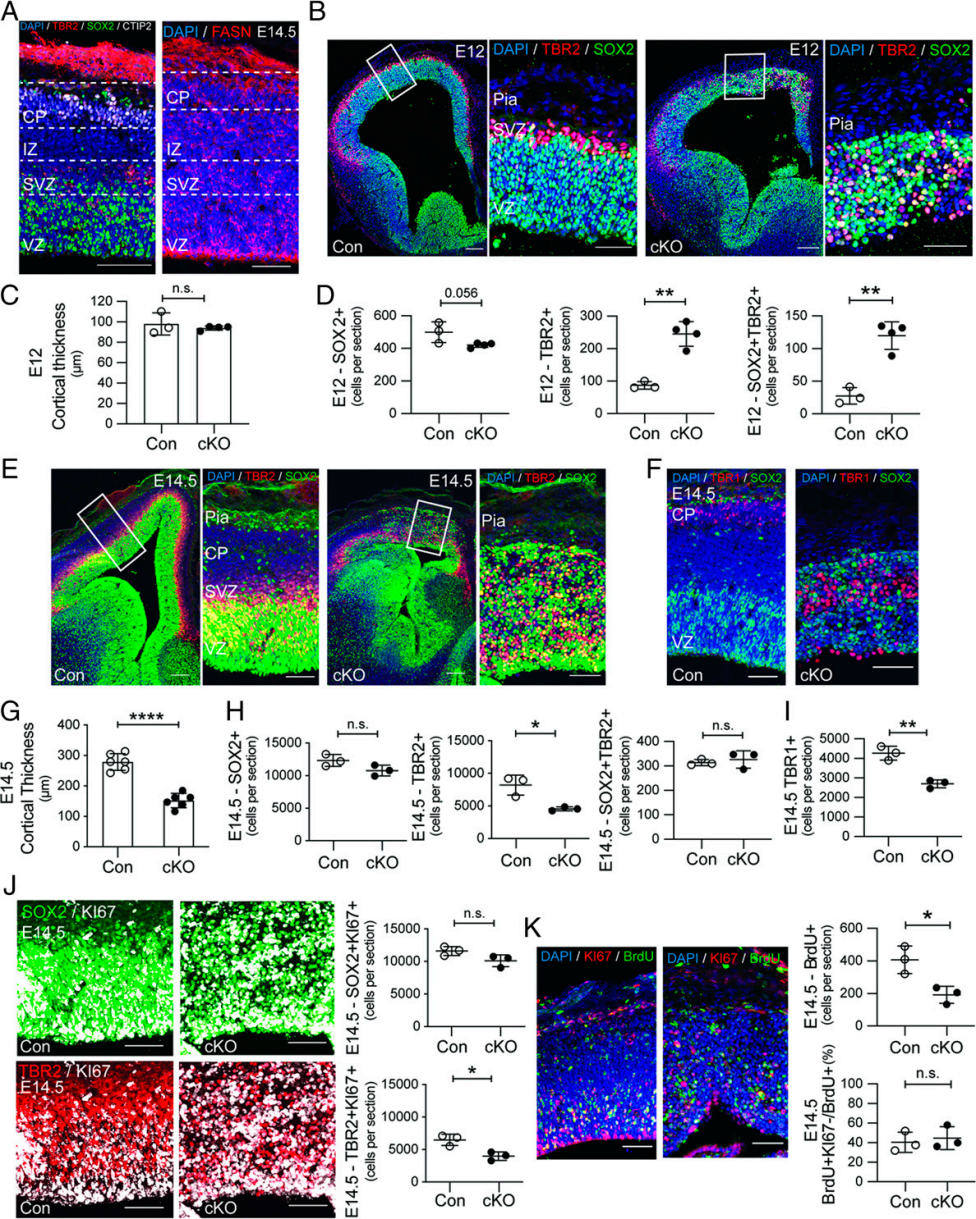
FASN is required for mouse cortex development. (*A*) Structure of the E14.5 developing mouse cortex with APs (SOX2, green), BPs (TBR2, red), and neurons (CTIP2, white). The right panel shows FASN expression (red) at E14.5. Dashed lines demarcate different layers of the developing cortex. (*B*) Compared to control (Con) mice (*Left*), FASN-cKO causes severe disorganization of the cortical wall at E12 and premature differentiation of SOX2-labeled progenitors (green) into TBR2-labeled (red) progenitors. Boxed areas are zoomed in. (*C*) The bar graph shows quantification of cortical thickness at E12. (*D*) Quantifications of the number of SOX2- and TBR2-labeled cells at E12. (*E*) At E14.5, FASN-cKO mice show microcephaly and disorganization within the cortical wall of SOX2-labeled (green) progenitors and TBR2-labeled (red) progenitors. Additionally, FASN-cKO mice show a reduction in number of TBR2-positive cells. Boxed areas are zoomed in. (*F*) TBR1-labeled neurons (red) are reduced and abnormally localized within the cortical wall compared to control (*Left*) at E14.5. APs are labeled with SOX2 (green). (*G*) The bar graph shows quantification of cortical thickness at E14.5. (*H*) Quantifications of the number of SOX2- and TBR2-labeled cells at E14.5. (*I*) Quantifications of TBR1-labeled neurons at E14.5. (*J*) FASN deletion causes reduced proliferation (KI67, white) of TBR2-labeled BPs (red) with SOX2-expressing (green) progenitor proliferation being not significantly affected. The bar graphs show quantifications of KI67-labeled cell-expression SOX2 (*Top*) and TBR2 (*Bottom*) at E14.5. (*K*) Retention of BrdU (green) is reduced in FASN-cKO mice 24 h after injection. However, cell-cycle exit and reentry, measured by BrdU and KI67 (red) colabeled cells, is not different between FASN-cKO and controls. The bar graphs show quantifications of BrdU (*Top*) and the percentage of BrdUKI67-labeled cells over total BrdU cells (*Bottom*) at E14.5. IZ, intermediate zone. Values are reported as mean ± SD; n.s., nonsignificant; *P < 0.05; **P < 0.005; and ****P < 0.0005 by unpaired *t* test; each data point depicts one embryo. (Scale bars, 100 µm in main panels and 50 μm in zoomed panels.)

Previous work showed that FASN is enriched in adult neural stem cells and is important for their proliferation (27, 28). Suggesting a role for FASN also during embryonic brain development, we found an enrichment of FASN in the VZ compared to upper regions in the embryonic pallium at embryonic day (E) 14.5 (Fig. 1*A* and *SI Appendix*, Fig. S1*A*). To test the functional relevance, and given that complete deletion of Fasn in mice leads to preimplantation embryonic death (32), we conditionally deleted Fasn in the embryonic telencephalon by crossing mice harboring floxed Fasn alleles (Fasn^flox/flox^) with Emx1^Cre^ mice (hereafter called Fasn-cKO). We confirmed the efficiency of genetic Fasn deletion by measuring FASN levels in the cortex at different embryonic time points using immunofluorescence. *Emx1* starts being expressed at E9.5 (33), but FASN protein levels were not yet reduced at E10.5 (*SI Appendix*, Fig. S1*A*). However, FASN protein was virtually absent at E12 and E14.5 in the pallium (*SI Appendix*, Fig. S1 *B* and *C*). Emx1Cre-mediated deletion of Fasn caused severe disorganization of cortical layers that lacked a clear separation between VZ, SVZ, and the cortical plate (CP) at E12 and E14.5, with cells of all differentiation stages (APs expressing SOX2, BPs expressing TBR2, and neurons expressing CTIP2 or TBR1) being aberrantly located throughout the pallial wall upon Fasn loss (Fig. 1 *B*, *E*, and *F*). To gain further insights into the microcephalic phenotype upon Fasn deletion, we analyzed the embryonic cortex at different developmental time points. While cortical thickness was not affected at E12, we found increased numbers of TBR2-labeled BPs and premature neuronal differentiation at E12; AP numbers and progenitor proliferation, measured using KI67, were not substantially affected (Fig. 1 *C* and *D* and *SI Appendix*, Fig. S1 *D* and *E*). However, cell death levels were increased at E12 (*SI Appendix*, Fig. S1 *F* and *G*). At E14.5, the AP progenitor pool and the number of BPs and neuronal cells (expressing TBR1) were reduced, causing a substantial reduction of cortical thickness in Fasn-cKO mice compared to controls (Fig. 1 *E*–*I*). BPs showed reduced proliferation at E14.5 (Fig. 1*J*). However, we found no differences in cell-cycle exit/reentry dynamics between progenitors in Fasn-cKO mice and controls (Fig. 1*K*). Thus, our data indicate that initial premature neuronal differentiation, reduced BP cell proliferation, and a loss of APs cause severe microcephaly in Fasn-cKO mice.

### FASN Is Required for AP Polarity.

**Fig. 2.**
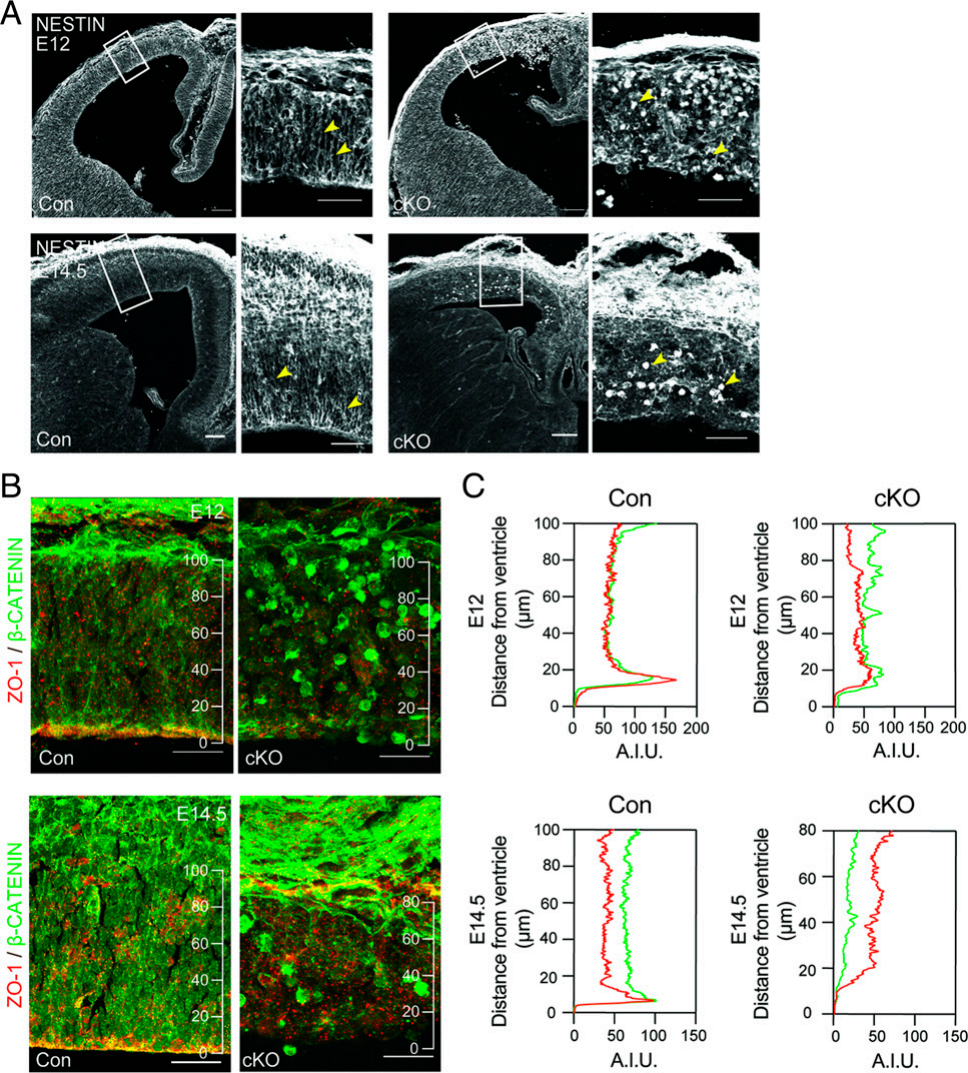
FASN deletion disrupts progenitor-cell polarity. (*A*) Analysis of the intermediate filament Nestin (white), labeling the radial glia scaffold, shows disorganization of the embryonic cortex at E12 (top row of panels) and E14.5 (bottom row of panels). Boxed areas are zoomed in. Arrowheads point toward radial processes extending from APs in control and collapsed process in Fasn-cKO animals. (Scale bars, 100 μm in main panels and 50 μm in zoomed panels.) (*B*) Expression of proteins associated with adherens junctions and cell polarity (ZO-1, red; β-catenin, green) shows disrupted polarity at E12 (*Top*) and E14.5 (*Bottom*) in the developing cortex. (*C*) Shown are intensity profiles (measured in A.I.U.; arbitrary intensity units) of ZO-1 and β-catenin throughout the cortical wall with distance from the ventricle. Note the enriched signal of ZO-1 and β-catenin in close proximity to the ventricle that is lost upon FASN deletion. (Scale bars, 25 µm.)

The establishment and maintenance of cellular polarity play a pivotal role for proper morphogenesis of the developing cortex (34, 35). The apico-basal domain is crucial for AP behavior, and radial process extending from APs toward the pia, forming a radial glia scaffold, are required for neuronal migration and correct cortical lamination. Given the observed laminar disorganization of the pallium upon Fasn deletion (Fig. 1 *B* and *E*), we hypothesized that cell polarity and maintenance of the apical domain may be affected in Fasn mutant progenitors. The radial glia scaffold, visualized using the intermediate filament Nestin, lacked the typical columnar organization and was instead collapsed in Fasn-cKO mice at E14.5 compared to controls (Fig. 2*A*). The integrity and extension of Nestin-labeled radial processes were affected at both apical and basal regions of the developing cortex in Fasn-cKO mice (Fig. 2*A* and *SI Appendix*, Fig. S2*D*). Furthermore, we analyzed the expression and localization of two markers of the apical domain, ZO-1 and β-catenin, which are both localized to adherens junctions of APs in the embryonic brain (36, 37). Instead of forming a defined apical domain, we found aberrant localizations of ZO-1 and β-catenin in Fasn-cKO mice, with a complete loss of tissue polarization (Fig. 2*B*). Thus, our data indicate that genetic deletion of de novo lipogenesis not only alters progenitor activity but also affects cellular polarity of progenitor cells in the developing mouse cortex.

### Pharmacological Inhibition of FASN Affects Integrity of Forebrain Organoids.

**Fig. 3.**
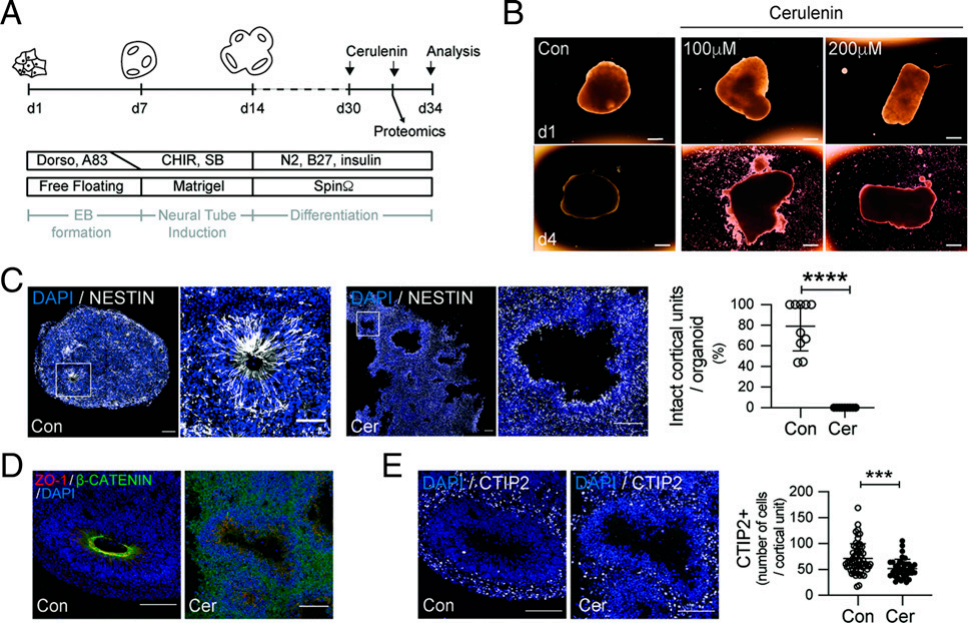
FASN regulates development of human forebrain organoids. (*A*) A schematic showing the strategy to generate hESC-derived forebrain organoids and experimental outline of FASN pharmacological inhibition using the FASN inhibitor Cer. (*B*) Bright-field images of d30 forebrain organoids after 4 d of treatment with vehicle (Con) or Cer. Note the disruption of tissue integrity upon FASN inhibition. (Scale bars, 1 mm.) (*C*) Radial glia scaffold (Nestin, white) assessment in cortical units from control and Cer-treated organoids. Boxed areas are zoomed in (*Right*). Quantification of the percentage of intact cortical units in analyzed organoids. (*D*) Cell-polarity (ZO-1, red; β-catenin, green; in lower panels) assessment in cortical units from control and Cer-treated organoids. (*E*) FASN inhibition causes reduced neurogenesis, as measured by quantifying the number of CTIP2-labeled neurons (white) per cortical unit. All analyses were done comparing control and Cer 200 µM. CHIR, CHIR-99021; EB, embryoid body; Dorso, dorsomorphin; SB, SB-431542. Values are reported as mean ± SD; ***P < 0.001 and ****P < 0.0005 by unpaired *t* test; each data point depicts an organoid in *C* and a cortical unit in *D*. (Scale bars, 100 µm in main panels and 50 µm in zoomed panels.)

After identifying a crucial role of proper FASN activity for cortical morphogenesis in the embryonic mouse brain, we next aimed to determine if FASN’s relevance for brain development is conserved in human tissues. Previous work identified high levels of *FASN* RNA expression in APs of the human developing cortex and showed that enhanced FASN activity, caused by a human variant associated with intellectual disability, affects progenitor-cell activity (11, 30). We probed the relevance of FASN in human progenitors by analyzing regionalized forebrain organoids derived from hESCs using a previously established approach (30, 38). We reduced FASN activity in 30-d-old organoids by using two pharmacological inhibitors of FASN, Orlistat and Cerulenin (Cer) (Fig. 3 *A* and *B* and *SI Appendix*, Fig. S3*A*) (39, 40). A total of 4 d after the addition of Cer, organoids with FASN inhibition showed reduced numbers of neurons and a substantially altered structural organization (Fig. 3 *B*–*E* and *SI Appendix*, Fig. S3*B*), which was associated with reduced mitotic activity within the pool of outer radial glia progenitors, visualized using phospho-Vimentin (pVim) and SOX2 (*SI Appendix*, Fig. S3*C*). Indeed, continuous spinning in bioreactors upon exposure to pharmacological FASN inhibitors caused a loss of structural integrity within organoids, which consist of several cortical units starting to break apart (Fig. 3 *B* and *C*). Corroborating the effects of Fasn deletion in mouse progenitors, we found that pharmacological inhibition of FASN caused a lack of apico-basal cell polarity, visualized by the localization of ZO-1 and β-catenin, a collapse of the radial glia scaffold, as measured by Nestin localization (Fig. 3 *C* and *D*). Thus, hESC-derived organoids show FASN dependency for proper organization and maintenance of structural integrity.

### Organoid Imaging Reveals Function of FASN for Polarity of Human APs.

**Fig. 4.**
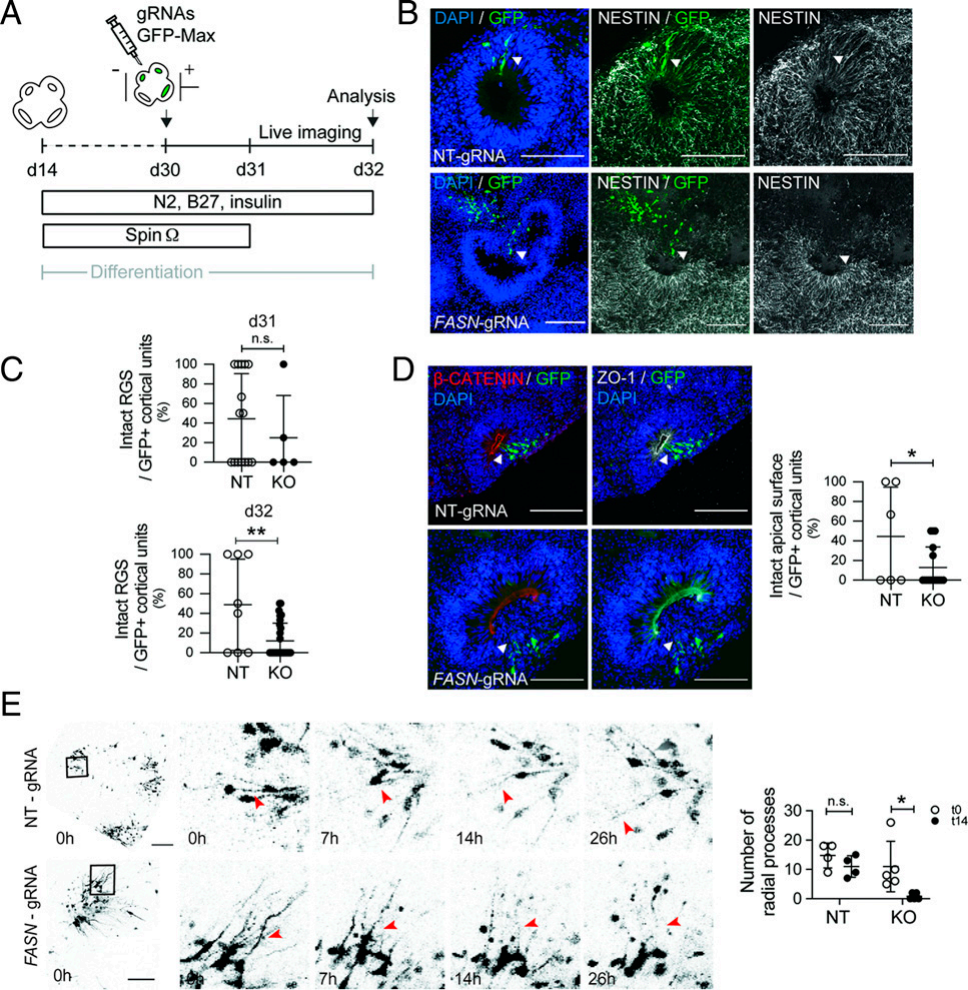
Genetic ablation of FASN in forebrain organoids affects polarity of human progenitors. (*A*) A schematic showing the design of in organoid electroporation and live-imaging experiments. (*B*) Radial glia scaffold (RGS; Nestin, white) integrity assessment in cortical units targeted with control or FASN-KO gRNAs (visualized using GFP, green). Note disruption of the radial scaffold in FASN-targeted areas. Arrowheads point toward examples of electroporated cells. (Scale bars, 50 µm.) (*C*) Quantification of the percentage of intact electroporated cortical units 24 h (d31) and 50 h (d32) after electroporation. (*D*) Cell-polarity (β-catenin, red; ZO-1, white) integrity assessment in cortical units targeted with control or FASN-KO gRNAs, revealing impaired integrity of cortical units upon FASN deletion. Arrowheads point toward examples of electroporated cells. (Scale bars, 50 µm.) (*E*) Time course of cortical units with GFP-labeled APs with visible radial processes and quantification of remaining processes after 26 h of imaging. Note the collapse of radial processes upon FASN deletion (red arrowheads) compared to electroporated control cells. Boxed areas are zoomed in (*Right*). The bar graph shows quantification of intact organoids at the beginning of imaging (t0 = 24 h after electroporation) and at the end of imaging (50 h after electroporation, 26 h after beginning of imaging). Arrowheads point toward radial processes from GFP+ cells. (Scale bars, 25 µm.) NT, nontargeting guide RNA; KO, FASN knock out guide RNAs. Values are reported as mean ± SD; n.s., not-significant; *P < 0.05; **P < 0.005; unpaired *t* test; each data point depicts an organoid in *C* and *D* and an imaged cortical unit in *E*.

Given the dramatic morphological changes in forebrain organoids with pharmacological inhibition of FASN, we next genetically deleted Fasn from neural progenitors within hESC-derived organoids using a CRISPR-Cas9–based approach (Fig. 4*A*). Guide RNAs (gRNAs) targeting the second exon of the human *FASN* gene were efficient to reduce FASN levels compared to nontargeting gRNA controls (*SI Appendix*, Fig. S4 *A*–*C*). Using in organoid electroporation of gRNAs, we targeted cells lining the ventricle-like regions from several cortical units within individual organoids at day 30. *FASN*-targeting gRNAs (or nontargeting control gRNAs) were coelectroporated with green fluorescent protein (GFP)-expressing plasmids, allowing for the visualization of electroporated cortical units. Analyzing cortical units 24 h after electroporation revealed that cortical units with genetic Fasn deletion were comparable to controls (Fig. 4*C*). However, 50 h after electroporation, Fasn-deleted cells showed a strongly disrupted radial morphology, as measured using Nestin, and aberrant cellular polarity, as measured using ZO-1 and β-catenin. (Fig. 4 *B*–*D*). In contrast, organoids that were electroporated with nontargeting control plasmids showed preserved radial glia scaffold morphology and intact apical domains (Fig. 4 *B*–*D*). These findings suggest that, analogous to mouse developing cortex, FASN-dependent lipogenesis is crucial for polarity of human neural progenitors.

To analyze the dynamics of how Fasn deletion affects AP polarity, we followed individual cells upon Fasn deletion using time-lapse microscopy in live organoids. Whereas the structure of radial processes was comparable between Fasn-deleted and control cells 24 h after electroporation, we found that apical processes extending from progenitors that had received Fasn-targeting gRNAs became highly unstable within 26 h of time-lapse imaging and were more likely to collapse compared to nontargeting control cells (Fig. 4*E* and Movies S1 and S2). Thus, our data reveal that FASN-dependent de novo lipogenesis is required for proper progenitor behavior in the developing brain and alters cellular polarity in mouse and human progenitor cells.

### FASN Inhibition Alters the Proteome of CD133-Labeled Progenitors.

**Fig. 5.**
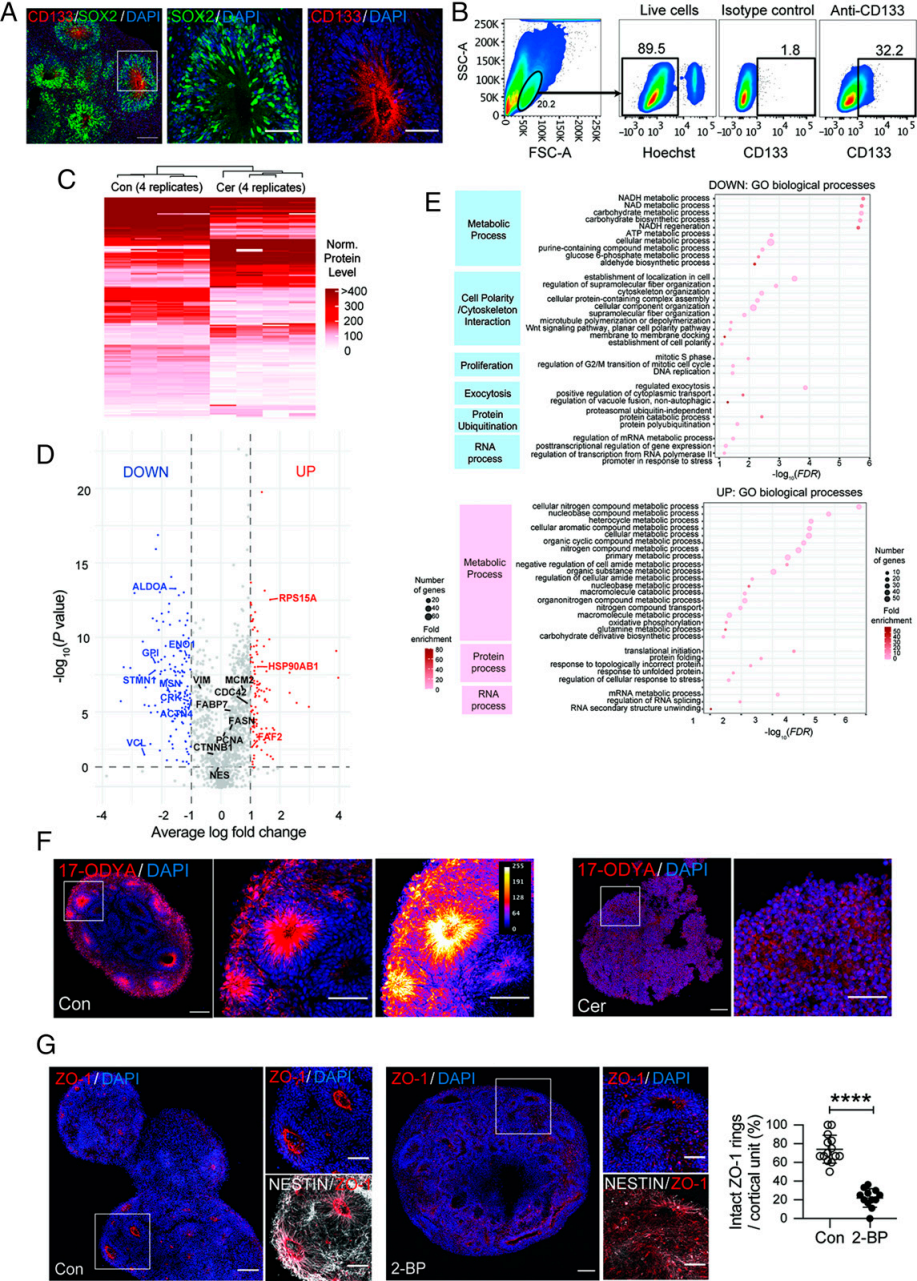
Inhibition of FASN alters the proteome of neural progenitors. (*A*) Immunofluorescent analyses showing the expression of CD133 (red) in human neural stem/progenitor cells (NSPCs) (labeled with SOX2 in green). (Scale bars, 50 µm.) (*B*) FACS plots showing CD133+ population within whole wild-type organoids. (*C*) Heatmap comparing differentially expressed proteins between control and Cer-treated CD133-sorted NSPCs from MS analysis. (*D*) Volcano plot showing differentially expressed proteins. (*E*) GO term selection (biological process) for down- and up-regulated proteins. Dot size represents the number of proteins included in the term; shading represents the fold enrichment compared to the whole proteome. Only GO terms with FDR (false discovery rate) <0.01 were selected and redundancy trimmed. (*F*) Enrichment of palmitoylated proteins in the apical domain of cortical units in control samples compared to Cer-treated organoids, as shown by 17-ODYA labeling (red). The right panel shows false-colored, scaled-intensity measurements. (Scale bars, 100 µm in main panels and 50 µm in zoomed panels.) (*G*) Inhibition of palmitoylation by 2-BP treatment causes loss of cell polarity, as measured by ZO-1 localization. (Scale bars, 100 µm in main panels and 50 µm in zoomed panels.) Values are reported as mean ± SD; ****P < 0.0005; unpaired *t* test; each data point depicts an organoid.

To start characterizing molecular alterations of human neural progenitors upon FASN inhibition, we analyzed the proteome of CD133-labeled cells. CD133 (also called Prominin-1) is a transmembrane glycoprotein that is enriched in mouse and human progenitors (41–44). We used low-input mass spectrometry (MS)-based proteomics of CD133-positive cells isolated by FACS in 30-d-old organoids and compared vehicle-treated control organoids to 24 h Cer-treated organoids that showed slight reductions of CD133 levels (Figs. 3 *A* and *B* and 5 *A* and *B* and *SI Appendix*, Fig. S5*A*). We identified 202 proteins showing differential levels (80 higher and 122 lower with Cer) (Fig. 5 *C* and *D* and Datasets S1 and S2). Interestingly, gene ontology (GO) enrichment analysis revealed down-regulation of proteins involved in cell polarity and cytoskeleton interaction (e.g., VCL, ACTN4, STMN1, CRK, and MSN), fully in line with the cellular phenotype of the altered polarity identified in mouse and human APs (Fig. 5*E*, *SI Appendix*, Fig. S5 *B* and *C*, and Datasets S3–S5). Furthermore, proteins annotated with cell proliferation and several metabolic processes, such as NADH (nicotinamide adenine dinucleotide reduced) and carbohydrate metabolism, were reduced (e.g., GPI, ALDOA, and ENO1), while proteins associated endoplasmic reticulum stress, unfolded protein response, and RNA processing were increased upon FASN inhibition (e.g., HSP90AB1, FAF2, and RPS15A) (Fig. 5*E*, *SI Appendix*, Fig. S5 *B* and *C*, and Datasets S3–S5). Thus, pharmacological inhibition of FASN leads to molecular alterations in human CD133-labeled cells that may underlie the polarity-associated neural progenitor phenotype. To substantiate a link between lipid metabolism and cell polarity, we hypothesized that polarity may depend on S-acylation (palmitoylation), a reversible lipid modification of proteins affecting the localization, stability, and protein interactions (45) of proteins involved in progenitor-cell polarity. Therefore, we assessed the presence of palmitoylated proteins using the palmitate analog 17-octadecynoic acid (17-ODYA) (46). Interestingly, we observed enriched levels of 17-ODYA–labeled proteins around the ventricular surface and within radial processes from APs, which were lost upon Cer treatment (Fig. 5*F*) (47). To determine if protein palmitoylation indeed affects cell polarity, we added 2-bromo-palmitate (2-BP), an inhibitor of protein palmitoylation (48, 49), for 4 d to forebrain organoids. Supporting a role of palmitoylation for cell polarity, 2-BP treatment substantially altered AP polarity, as measured by a lack of a clear ZO-1 apical belt in a dose-dependent manner (Fig. 5*G* and *SI Appendix*, Fig. S5*D*). However, 2-BP–treated organoids did not show a complete loss of radial processes, indicating that the effects of *FASN* deletion do not exclusively depend on a reduction of protein palmitoylation (Fig. 5*G*).

## Discussion

We here used conditional gene deletion and pharmacological inhibition to identify a role for FASN-dependent de novo lipogenesis for mouse and human brain development. Impaired FASN activity is linked to impaired cell proliferation and disturbs polarity of progenitor cells that is required to maintain the structural integrity of the developing cortex.

Cellular metabolism has been recently identified to be of pivotal importance for proper embryonic development, sensing environmental conditions (e.g., availability of substrates), and regulating virtually all steps from cell proliferation, fate choice decisions, and subsequent differentiation and integration of daughter cells (19, 20). Proliferating neural progenitors show high levels of aerobic glycolysis before they switch upon differentiation to OXPHOS, fueling the TCA cycle for energy production (21–24, 50). In addition, a variety of other metabolic pathways, including glutaminolysis, 1-carbon metabolism, and cholesterol metabolism, are required for normal brain development (26, 51–53). Catabolic lipid metabolism (i.e., the breakdown of fatty acids via β-oxidation) has been shown to be important for symmetric, duplicating progenitor-cell divisions in the embryonic brain (24, 25). Supporting the relevance of cellular metabolism for brain development, a number of variants and mutations in enzymes involved in the regulation or execution of metabolic pathways have been linked to genetic diseases affecting the brain (19).

However, a role for anabolic lipid metabolism (i.e., the conversion of glucose into fatty acid mediated through FASN) in the context of cortex development remained unexplored. Previous work showed that progenitors in the adult brain require FASN for proliferation and that a human point variant, causing enhanced FASN activity, impairs human progenitor proliferation (27, 28, 30). Moreover, several recently identified de novo mutations in human *FASN* have been associated with developmental epileptic encephalopathy, further indicating relevance of FASN for human brain development and disease (31). Using genetics and pharmacological inhibition, we here establish a role for de novo lipogenesis for mouse and human neural progenitor function.

How does FASN exert its function on brain development? Whereas basic metabolic shifts in the course of neurogenesis are beginning to be understood, the exact cell state–dependent changes for major metabolic pathways, from duplicating APs, to asymmetrically dividing APs, to proliferating BPs, and eventually to differentiating neurons, remain obscure (19). This is due to the fact that most metabolic analysis, such as metabolomics or metabolite tracing, relies on relatively large amounts of starting tissues, thus making analyses with high spatial (e.g., cellular) and temporal resolution challenging (54, 55). Single-cell genomics, increasingly obtained in mouse and human tissues, is helpful to delineate metabolic programs (11, 16, 42, 56, 57). However, it is also clear that metabolism is not only regulated via gene transcription but rather is a highly complex process that depends on substrate availability and localization/availability of rate-limiting enzymes (58). Thus, future work will require advanced technology allowing for zooming in onto each developmental step from progenitor expansion to neuronal differentiation. Indeed, metabolic adaptations may serve as an integration hub of how a plethora of signaling pathways, including bone morphogenic protein (BMP) or WNT signaling, both associated with regulating metabolism, will ultimately affect cellular behavior and fate in the developing brain (59, 60). Effects of metabolism on cell behavior may be achieved through providing sufficient energy and supplying building blocks for cell growth but also by regulating other cellular processes such as RNA transcription (19, 20, 61).

The finding that AP polarity is altered by FASN inhibition provides a link between metabolism and cell polarity in mouse and human progenitors. Proteomics of CD133-labeled progenitors revealed, among other changes, reduced levels of proteins annotated in cell polarity and cytoskeletal interactions, further supporting a role for lipid metabolism in neural progenitor-cell polarity. The functional relevance of differentially regulated proteins upon FASN inhibition needs to be tested in future work. However, it is tempting to speculate that, beyond a potential role of lipogenesis for cell growth, an FASN-dependent supply of palmitate may be required for palmitoylation (also referred to as S-acylation) and, therefore, correct function of components of the apical domain in APs. Palmitoylation is a reversible lipid modification of proteins, affecting the localization, stability, trafficking, and protein interactions of lipid-modified proteins (45). Indeed, previous work showed that the depalmitoylase APT1 is important for asymmetric partitioning of Notch and WNT signaling components during asymmetric cell division (62). In addition, other proteins involved in progenitor-cell polarity, such as CDC42 and JAM-3, have been shown to be palmitoylated (48, 63–65). Notably, FASN-dependent palmitoylation has been identified to be important for S-acylation of proteins, such as MUCIN-2 and eNOS, outside the brain (66, 67). Indeed, we provide evidence for an enrichment of palmitoylated proteins, as measured by 17-OYDA incorporation, in apical domains and radial processes of APs. Furthermore, integrity of cortical units was affected by 2-BP–mediated inhibition of palmitoylation. Given that neural progenitors show a large number of palmitoylated proteins and that palmitoylation of, for example, BMP receptors is important to guide the fate of embryonic progenitors (68), future work will be needed to determine if the palmitoyl proteome is affected in APs upon manipulation of FASN levels.

The data shown here establish a critical role for FASN-mediated lipogenesis for mouse and human progenitor behavior and provide a link between lipid-dependent cellular metabolism and cell polarity in the developing cortex.

## Materials and Methods

### FASN-cKO Animal Model and Handling.

All experimental animals were Fasn^flox/flox^;Emx1^Cre/+^ and compared to Fasn^flox/WT^;Emx1^+/+^ littermate controls (33, 69). For timed pregnancies, the date of plug was defined as E0.5 and a lethal dose of anesthesia (Esconarkon, Streuli) was given intraperitoneally to the pregnant dam before embryo collection. For cell-cycle exit experiments, pregnant dams were injected with BrdU (50 mg/kg) intraperitoneally at E13.5 and embryos were collected 24 h later. Mice were kept in ventilated cages under a 12-h dark/light cycle with access to food and water. All animal experiments were performed in accordance with Swiss regulatory standards and approved by the veterinary office of the Canton of Zurich.

### hESC and Organoid Cultures.

H9-hESCs (Wicell, passage 31) were cultured as described in ref. 30. For electroporations, cells were kept for at least 2 h in 10 µM Y-27632, followed by single-cell passage using Accutase (Sigma-Aldrich). Approximately 2 million cells were electroporated using the Nucleofector Kit V (Lonza) in an AMAXA electroporator using program A-23. Afterward, cells were plated on glass coverslips and kept for 24 h in mTeSR Plus containing 10 µM Y-27632. All experiments done using hESCs were approved by the ethics commission of the Canton of Zurich, Switzerland. Organoids were generated as described before with slight modifications as described in *SI Appendix*, *Supplementary Materials and Methods* (30, 38). Forebrain organoid tissue was prepared by fixing in 4% paraformaldehyde/0.1 M phosphate buffer for 30 min at room temperature, followed overnight by 30% sucrose/PBS (phosphate buffer saline) at 4 °C. Fixed tissue was then embedded in cryoprotectant O.C.T. (Sakura), snap frozen in liquid nitrogen, and stored at −20 °C before sectioning.

### FASN Pharmacological Inhibition, Palmitate Labeling, and Palmitoylation Inhibition Experiments.

For FASN pharmacological inhibition experiments 1 through 3, organoids were placed in eight-well chamber slides (Nunc-Lab Tek, Thermo Fisher) and fed with media supplemented with the indicated concentration of Cer or Orlistat, replaced every 24 h. Control wells were supplemented with EtOH (ethanol) and DMSO (dimethylsulfoxide), respectively. For proteomic experiments, 35-d-old organoids were treated with Cer for 48 h and were further dissociated using a previously described protocol and FACS (fluorescence activated cell sorting) sorted using an APC (allophycocyanin)-coupled anti-CD133 antibody (70). For palmitate labeling, organoids were treated with 20 µM 17-ODYA (Sigma-Aldrich) 4 h before fixing. 17-ODYA fluorescent labeling was done using a click-chemistry reaction kit (Invitrogen) according to the manufacturer’s instructions. For the palmitoylation inhibition experiment, organoids were kept in 2-BP for 4 d before collection.

### In Organoid Electroporation.

A solution containing the gRNAs (2 µg each), GFP-Max reporter (4 µg) plasmids, and Fast Green (diluted 1:100 in water) was injected into the organoids using a FemtoJet 4i microinjector (Eppendorf) and a sharpened glass capillary needle. Injected organoids were imbibed in nucleofector solution and electroporated using the same protocol as for hESCs. Organoids were then washed and placed in spinners for 24 h. Only the organoids that maintained macroscopic integrity were kept for live imaging or tissue processing. For live-imaging experiments, two organoids were placed in four-well chamber slides (Nunc-Lab Tek, Thermo Fisher) and imaged for 26 h.

### Immunohistochemistry/Immunocytochemistry.

Embryo heads were dissected on ice-cold PBS and placed in 4% paraformaldehyde/0.1 M phosphate buffer overnight at 4 °C, followed overnight by 15% and subsequent 30% sucrose/PBS at 4 °C. Fixed heads were then embedded in cryoprotectant Tissue-Tek O.C.T (Sakura), snap frozen in liquid nitrogen, and stored at −20 °C before sectioning. The 20-µm-thick mounted sections were obtained for histological analysis using a Cryostat (Leica). Both embryonic mouse and organoid frozen tissue were sectioned using a Cryostat (Leica), and 20-µm-thick sections were obtained for histological analysis. Samples were washed and blocked in PBS with 3% donkey serum (Millipore) and 0.25% Triton-X for 30 min prior to antibody incubation. Slides with primary antibody were kept at 4 °C for 24 to 48 h. Tissue was further washed and blocked again for 15 min followed by secondary antibody staining at room temperature for 1.5 h. For BrdU stains, mouse embryonic tissues were treated with 1 N HCl at 4 °C for 10 min, 2 N HCl at 37 °C for 30 min, and 0.1 M borate buffer (pH 8.8) for 10 min, followed by extensive washes with PBS. DAPI or Hoechst was used to counterstain cell nuclei. Tissues were coverslipped with ImmuMount (Thermo Fisher) and kept at 4 °C until imaged. Antibodies and dilutions used are outlined in *SI Appendix*, Table S2.

### Microscopy and Image Analysis.

Confocal imaging of embryonic tissue and cerebral organoids was done using a Zeiss LSM800. Bright-field images of organoids were taken with an Evos XL Core microscope (Life Technologies). Bright-field imaging of postnatal mouse brains was done using the SZ61 Stereo Microscope (Olympus), and immunofluorescent imaging was performed with a Slide Scanner AxioScan.Z1 (Zeiss). All image analyses were performed using Fiji (71). Further image analysis is described in *SI Appendix*, *Supplementary Materials and Methods*.

### Proteomics Sample Preparation and MS.

Upon organoid dissociation, CD133+ 500 cells/well were FACS sorted with a “single-cell” mask into a 384-well plate with water, sealed, and stored at −80 °C. For cell lysis, the plate was placed for 10 min in a PCR thermocycler at 95 °C (72). Samples were cooled down and 1 μL 100 ng/μL Trypsin solubilized in 500 mM Hepes buffer (pH 8.2) was added to the samples and incubated overnight at 37 °C. Enzymatic digestion was stopped by adding 4 µL 4% aqueous formic acid. Samples were spiked with iRT standard peptides (Biognosys) and loaded on Evotips following the provided instructions (Evosep). MS analyses were performed on an Evosep One (Evosep) coupled to the timsTOF Pro (Bruker), and experimental conditions are described in detail in *SI Appendix*, *Supplementary Materials and Methods*.

### Protein Quantification and Proteomics Analysis.

Protein quantification was performed with Spectronaut (Biognosys, version 14.9). Protein quantification is described in *SI Appendix*, *Supplementary Materials and Methods*. Only proteins present in at least three replicates were considered for the differential expression analysis. Proteins with at least a twofold change and *P* value below 0.05 were deemed significantly different between conditions. Differentially expressed proteins were then tested for overrepresentation of GO terms.

### Statistical Analysis.

Statistical analyses were done using Prism 9 (GraphPad). For two group comparisons, a two-tailed Student’s *t* test was used. For all experiments, all animals from the correct genotype from the litter and all organoids in the batch were analyzed. Proteomics data from control and Cer-treated organoids are available at the PRIDE (Proteomics Identification Database) database (identifier PXD026110) (73). All proteomics measurements (in quadruplicate) and differentially expressed protein quantification can be found in Datasets S1 and S2. KEEG pathways and GO terms over-represented in differentially expressed proteins can be found in *SI Appendix*, Table S1 and Datasets S3–S5.

## Supplementary Material

Supplementary File

Supplementary File

Supplementary File

Supplementary File

Supplementary File

Supplementary File

Supplementary File

Supplementary File

## Data Availability

Proteomics data have been deposited in the publicly accessible database PRIDE and assigned the identifier PXD026110. All other study data are included in the article and/or supporting information.
