# Supplementary material for: FASN-dependent de novo lipogenesis is required for brain development
Source: Proc Natl Acad Sci U S A. 2022 Jan 7;119(2):e2112040119. doi: 10.1073/pnas.2112040119 (PMC8764667; doi:10.1073/pnas.2112040119)
Supplement: Supplementary File [file pnas.2112040119.sapp.pdf]

Supplementary Information for

FASN-dependent *de novo* lipogenesis is required for brain development

Daniel Gonzalez-Bohorquez[1], Isabel M.G. López[1], Baptiste N. Jaeger[1], Sibylle Pfammatter[2], Megan Bowers[1], Clay F. Semenkovich[3], Sebastian Jessberger[1#]

[1]Laboratory of Neural Plasticity, Faculties of Medicine and Science, Brain Research Institute, University of Zurich, 8057 Zurich, Switzerland. [2]Functional Genomics Center Zurich, University of Zurich and ETH Zurich, 8057 Zurich, Switzerland. [3]Washington University School of Medicine, Division of Endocrinology, Metabolism & Lipid Research, St. Louis, USA
[#]Correspondence should be addressed to S.J.
Email: jessberger@hifo.uzh.ch

Supplementary Information includes:

Figures S1 to S5

Supplementary Materials and Methods

Legends for Tables S1 to S2

Legends for Datasets S1 to S5

Legends for Movies S1 to S4

Supplementary References

Other supplementary materials for this manuscript include:

Movies S1 – S4

Tables S1 – S2

Supplementary Figures

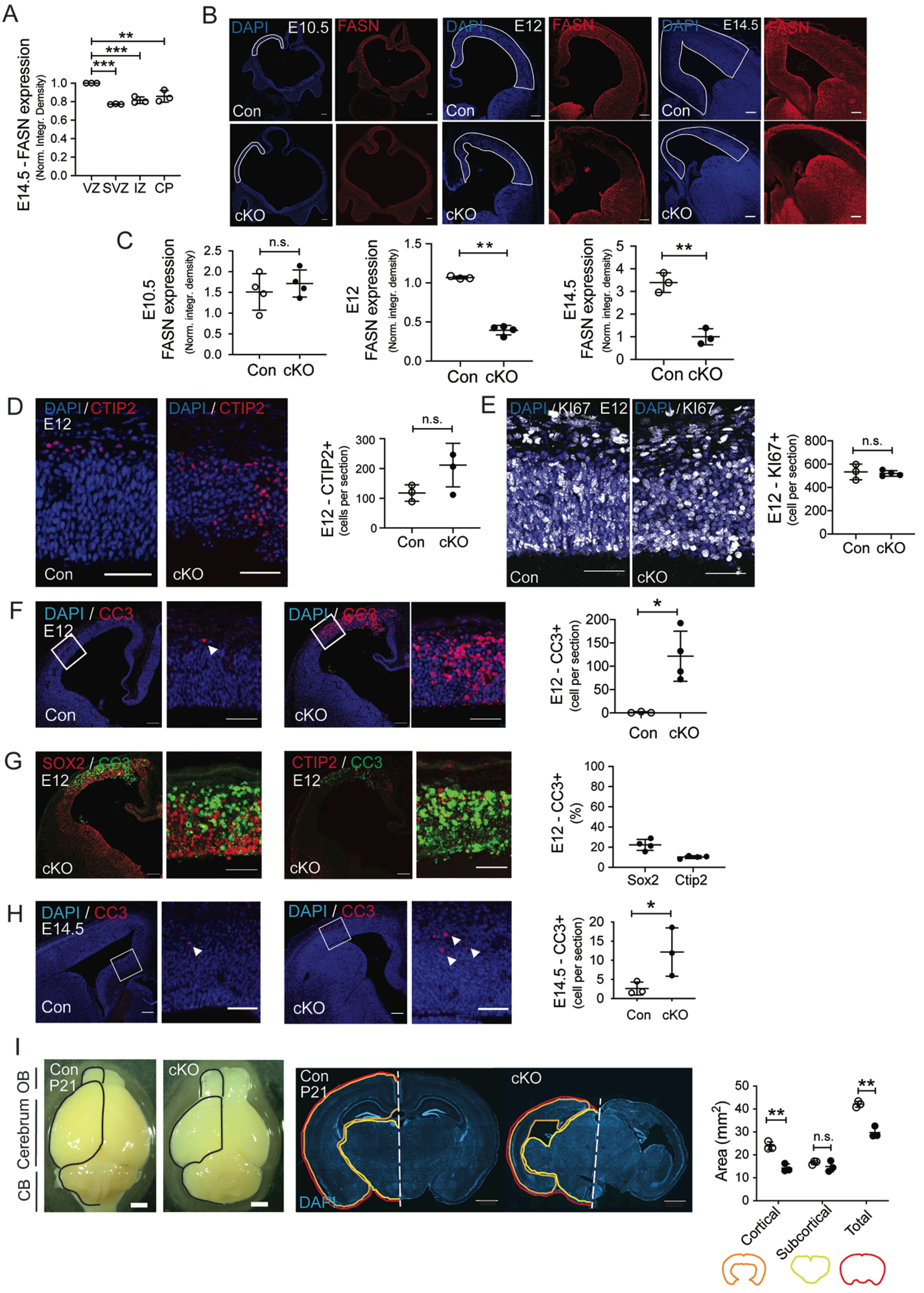

Figure S1. FASN expression is effectively reduced in cKO animals.

(A) Intensity quantification of FASN protein levels, revealing enrichment of FASN in the ventricular zone compared to upper regions of the developing cortex. Area quantified is outlined dashed line.

(B) Conditional deletion of FASN (red) using Emx1Cre-mediated recombination causes loss of FASN protein in the forebrain at E12 and E14.5 but not at E10.

(C) Graphs shows intensity quantification of FASN levels, normalized to FASN in the ganglionic eminences at E12 and E14 and to spinal cord at E10.

(D) CTIP2-labeled neurons (red) are abnormally localized within the developing cortex compared to controls. Quantifications of CTIP2-labeled cells at E12.

(E) Numbers of KI67-expressing, proliferating cells (white) are not affected by deletion of FASN at E12. Bar graphs show quantifications of KI67-labeled cells at E12.

(F) FASN deletion causes increased cell death (CC3, in red) in the developing forebrain at E12 in FASN-cKO animals (right panels) compared to control (left panel). Arrowheads point towards examples of CC3+ cells. Graphs show quantification of number of dying cells.

(G) Phenotyping of CC3 labelled cells reveals a higher percentage of dying apical progenitors (SOX2, labelled in red, right panel) compared to neurons (CTIP2, in red, right panels).

(H) FASN deletion causes increased cell death (CC3, in red) in the developing forebrain at E14.5 in FASN-cKO animals (right panel) compared to control (left panel). Arrowheads point towards examples of CC3+ cells. Graphs show quantification of number of dying cells.

(I) At P21, FASN-cKO mice show microcephaly. Cortical area quantification reveals that defects are restricted to the cortex. Scale bars represent 1 mm.

CC3, cleaved caspase 3; CP, cortical plate; IZ, intermediate zone; SVZ, sub-ventricular zone; VZ, ventricular zone. Values are reported as mean +/- SD, n.s, non-significant, *$p < 0.05$; **$p < 0.005$; ***$p < 0.001$, unpaired t-test, each datapoint depicts one embryo. Scale bars represent 100 µm in main panels and 50 µm in zoomed panels.

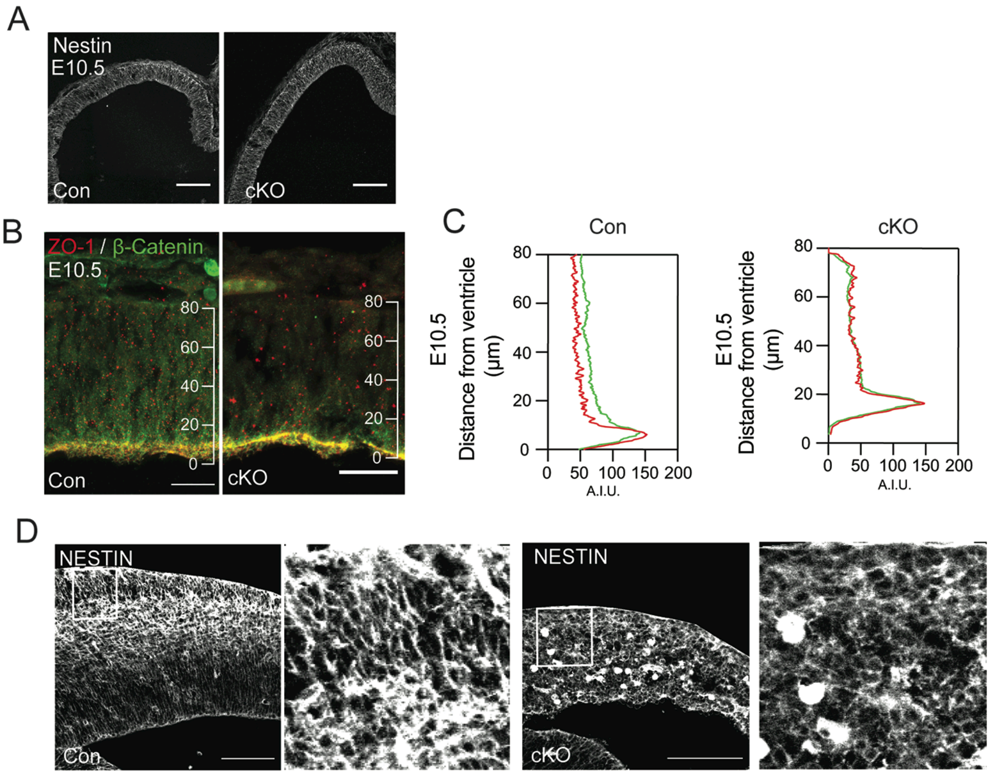

Figure S2. FASN-cKO animals show no defect in cell polarity at E10.

(A) Analysis of the intermediate filament Nestin (white), labeling the radial glia scaffold, shows no disorganization of the embryonic cortex at E10.

(B) Expression of proteins associated with adherens junctions and cell polarity (ZO-1, red; β-catenin, green) shows no disruption in cell polarity polarity at E10 in the developing cortex.

(C) Shown are intensity profiles of ZO-1 and β-catenin throughout the cortical wall with distance from the ventricle.

Scale bars represent 50 mm.

(D) Exemplary images showcasing the aberrant radial process morphology in the basal surface of the developing cortex. Smaller panels are zoomed in regions; to highlight Nestin-labelled process morphology changes (left-top). Scale bars represent 100 µm.

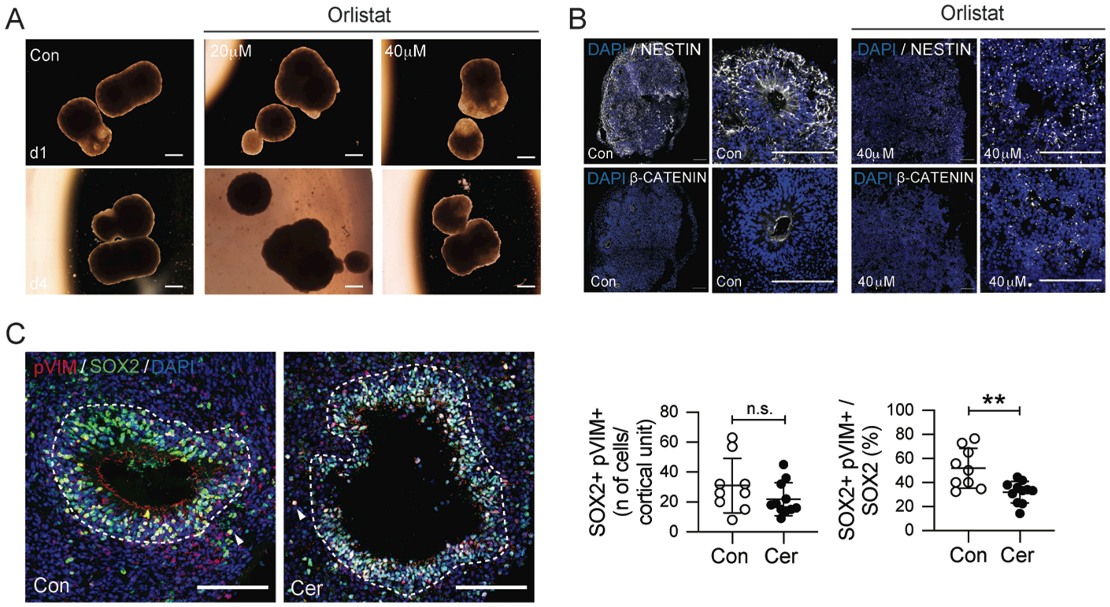

Figure S3. FASN inhibition with Orlistat affects forebrain organoid development.

(A) Bright-field images of d30 forebrain organoids after 4 days of treatment with vehicle (Con) or Orlistat. Note the disruption of tissue integrity upon FASN inhibition. Scale bars represent 1mm.

(B) Radial glia scaffold (Nestin, white) assessment in cortical units from control and 400µM Orlistat-treated organoids. Boxed areas are zoomed in (right panels).

(C) FASN inhibition causes no change in the total number of basal radial glia cells (SOX2 and p-VIM labeled) outside of the dashed line, delineating the zone of densely packed SOX2-labeled cells within cortical units. Basal radial glia cells from Cer treated organoids show lower rates of proliferation within the pool of radial glia progenitors. White arrowheads point at SOX2 and p-VIM positive cells. Values are reported as mean +/- SD, ***p* < 0.005, unpaired t-test, each data point depicts a cortical unit.

Scale bars represent 100 µm in main panels and 50 µm in zoomed panels.

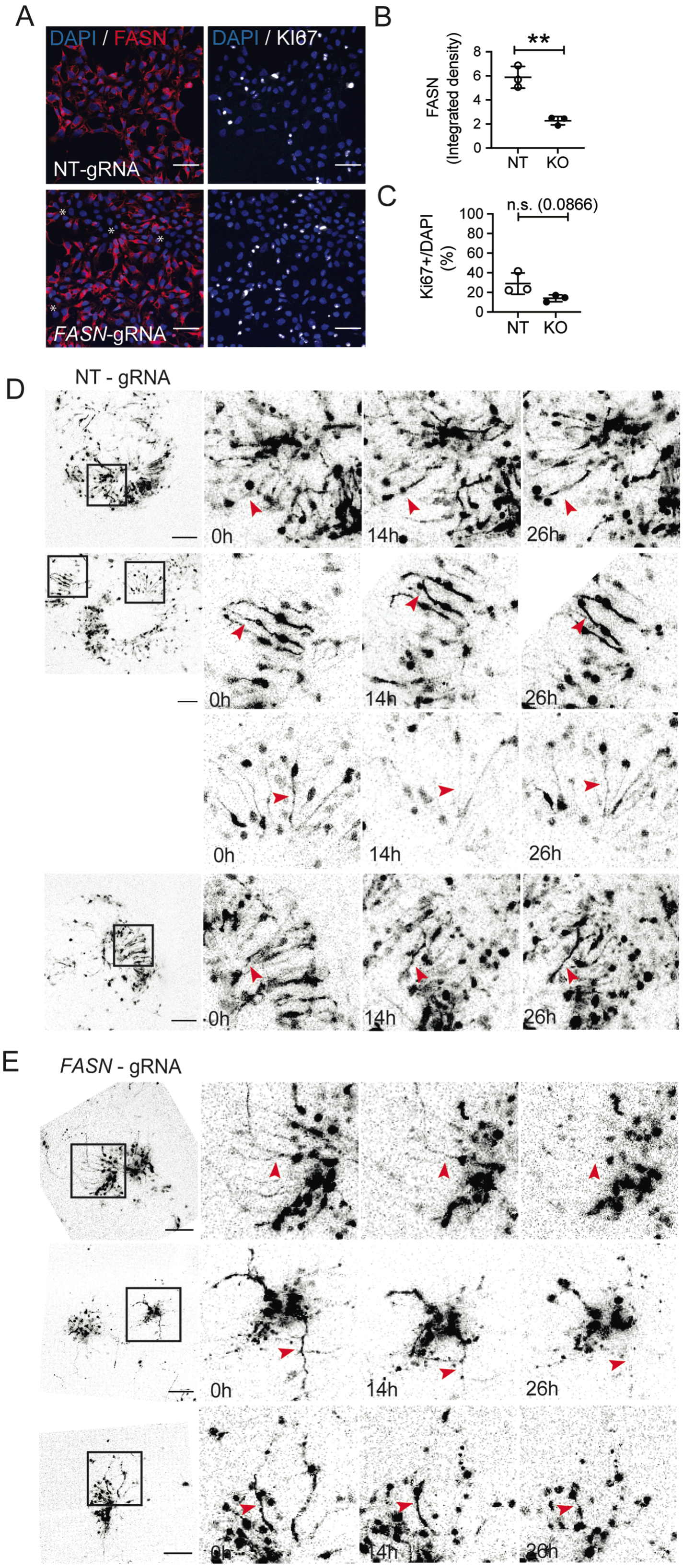

Figure S4. Genetic ablation of FASN in human embryonic stem cells.

(A) FASN (in red) expression levels were reduced in human embryonic stem cells electroporated with targeting gRNAs compared to non-targeting controls

(bottom panel) 48h after electroporation. Cycling cells are labeled with Ki67 (in white). Scale bars represent 25 μm

(B) Quantification of FASN levels in human embryonic stem cells 48h after electroporation.

(C) Percentage of Ki67 labelled cells in control cells and upon FASN deletion. Note the trend towards reduced levels of proliferation upon FASN deletion (p value is shown).

(D) Additional examples of time courses of cortical units with GFP-labelled apical progenitors with visible radial processes after 26h of imaging (red arrowheads) upon NT-gRNA electroporation. Boxed areas are zoomed in (right panels). Scale bars represent 50 μm.

(E) Additional examples of time courses of cortical units with GFP-labelled apical progenitors with visible radial processes after 26h of imaging (red arrowheads) upon *FASN*-gRNA electroporation. Boxed areas are zoomed in (right panels). Scale bars represent 50 μm.

gRNA, guide-RNA; NT, non-targetting. Values are reported as mean +/- SD, n.s, not-significant; ** $p < 0.005$; unpaired t-test, each data point represents a separate electroporation.

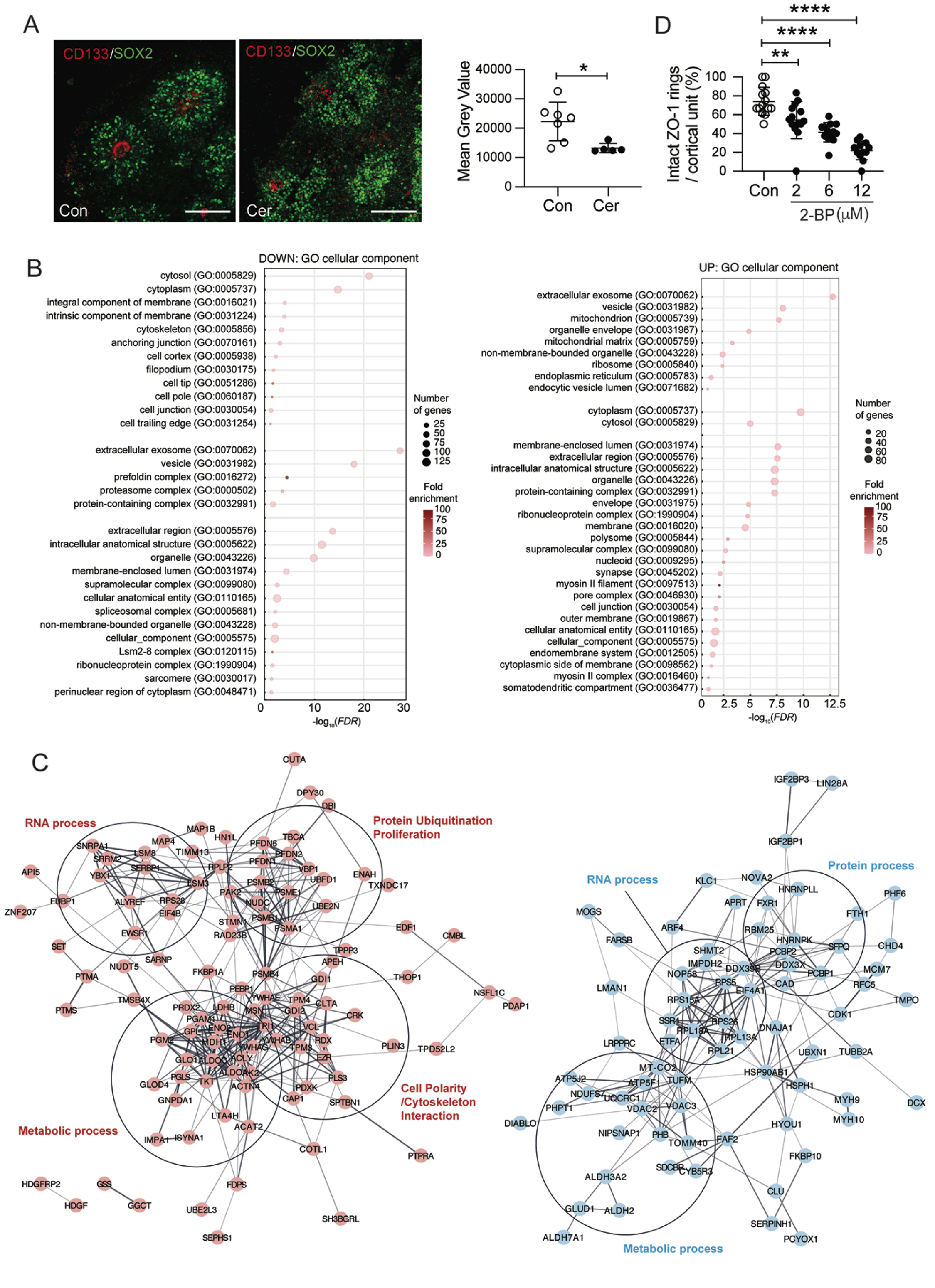

Figure S5. Proteome changes of neural progenitors upon FASN inhibition.

(A) FASN inhibition by Cerulenin treatment causes a reduction in the levels of CD133. Localization of the protein is still restricted to the center of the cortical units (labelled with SOX2). Values are reported as mean +/- SD, *$p < 0.05$;

unpaired t-test, each data point represents a cortical unit. Scale bars represent 50 µm.

(B) GO term selection (Cellular Component) for DOWN and UP-regulated proteins. Dot size represents the number of proteins included in the term; shading represents the fold enrichment compared to the whole proteome. Only GO terms with FDR < 0.01 were selected and redundancy trimmed using REVIGO.

(C) STRING analyses of protein association networks of DOWN and UP-regulated proteins after FASN inhibition.

(D) 2-BP treatment causes dose-dependent changes in the percentage of cortical units with ZO-1-labeled, dense apical domains. Values are reported as mean +/- SD, ***p* < 0.005; *****p* < 0.0001, unpaired t-test, each data point depicts one organoid.

Supplementary Materials and Methods

FASN-cKO animal model and handling

To generate $Fasn^{flox/flox}$;$Emx1^{Cre/+}$ embryos, $Fasn^{fl/+}$;$Emx1^{Cre/+}$ males were mated with $Fasn^{flox/flox}$ females (1, 2). For embryo collection a lethal dose of anesthesia (Esconarkon, Streuli) was given intra-peritoneally to the pregnant dam and upon lack of pain reflex from paw-pressing, dams were cervically dislocated and pups were extracted.

hESC culture

H9-hESCs were cultured in standard 37ºC and 5% CO_2 conditions in feeder-free conditions on hESC-grade Matrigel (Corning) coated plates (3). Cells were maintained in mTeSR Plus (Stem Cell Technologies) in the absence of antibiotics and media was changed every two days (4, 5). Cells used in all experiments were not kept further than 6 passages in culture. For culture maintenance cells were aggregate-passaged with ReLeSR (Stem Cell Technologies) to select for undifferentiated cells. After passaging cells were kept for 24h in media with 10μM Y-27632 (Stem Cell Technologies) to promote survival and stemness. hESCs were stored long-term as aggregates in CryoStore CS10 (Sigma-Aldrich) below -170ºC.

Forebrain-directed Brain Organoid generation

Organoids were prepared according to previously published protocols with some minor modifications (4). In brief, half a million hESCs were passaged into an AggreWell-800 well (24 well plate, Stem Cell Technologies) pre-treated with Anti-Adherence Rinsing Solution (Stem Cell Technologies). Cells were kept 24h in mTeSR Plus with 10μM Y-27632 until they formed embryoid bodies (EBs). EBs were collected the next day (day 1) and transferred to Ultra-Low Attachment Plates (Sigma-Aldrich) with TeSR-E5 (Stem Cell Technologies) supplemented with 2μM Dorsomorphin (Sigma-Aldrich) and 2μM A83-01 (Tocris) and fed on day 3. On day 4 and day 5, EBs were adapted to Induction media (TeSR-E5 supplemented with 1 μM CHIR990211, Stem Cell Technologies; and 1 μM SB431542, Stem Cell Technologies) performing half-volume media changes. On day 7 EBs were embedded in

growth factor reduced Matrigel (Corning) to induce neural tube formation. From day 7 to day 14 organoids were fed Induction media every two days. Finally, organoids were removed from the matrigel and placed in miniaturized spinning bioreactors at 100rpm until they were used for experiments between day 30 and day 40. During this time organoids were fed every two days with Differentiation media (DMEM-F12 supplemented with N2, B27, penicillin, streptomycin, NEAA, 100 µM β-mercaptoethanol, and 2.5 µg/mL insulin) and were monitored closely for integrity and media consumption. For live imaging experiments, two organoids were placed in 4 well chamber-slides (Nunc-Lab Tek, Thermo Fisher) and were imaged for 26h.

gRNA design and generation

Two gRNAs targeting Exon 2 of *FASN* in opposite directions (1 FW: CACCGTCAC GGA CGA TGA CCG TCG C; 2 FW: CAC CGG TGG TGA TTG CCG GCA TGT C) were cloned into a Cas9 plasmid with a puromycin resistance gene obtained from Addgene (Plasmid #62988) (6). In addition, a non-targeting gRNA was used as control (FW: CAC CGG TAT TAC TGA TAT TGG TGG G).

Organoid dissociation and CD133 staining for proteomics

Organoids were dissociated using a previously described protocol (7). Briefly, organoids were incubated in a solution of Accutase (Sigma Aldrich) and DNAse at 37ºC for 45min with intermittent low speed vortexing and pipetting with a P200 micropipette every 5-10min. Single cells were then pelleted by centrifugation (200g, 4min) and washed once with chilled DPBS. Cells were resuspended in DPBS with 1mM EDTA and strained prior to immunofluorescence staining. Afterwards, cells were stained at 4ºC in the dark for 30min with APC coupled anti-CD133 antibody (see Table S2). Finally, cells were washed once and resuspended in a solution of DPBS with Hoechst. Cells were sorted kept on ice before being sorted on a FACSAria III sorter (BD Biosciences).

Image analysis

All tiled images were stitched using ZEN Blue Software (Carl Zeiss). Cell counts were performed using Cell counter plug-in. Live imaging videos were compiled using the plug-in TrakEM2 (8). Experimenters were blinded for all quantifications. In the case of organoid analyses, experimenters were additionally blinded at the image acquisition step and all organoids contained within a slide were considered. For mouse embryonic sections cells were counted in three equivalent regions of anterior, middle, and posterior areas of the dorsal developing neocortex from 3-4 animals. Results were then averaged and each mouse was considered a statistical unit. For cell polarity analysis the function *plot profile* from Fiji was used using a 100 µm wide line. For organoid image analysis only cortical units that had a clear Sox2+ labelled ring were considered for the analyses. In the case of electroporated organoids only the cortical units that had GFP+ cells were considered. For organoid quantifications each cortical unit was considered as a statistical unit, unless otherwise specified.

Mass Spectrometry

MS analyses were performed on an Evosep One (Evosep) coupled to the TIMS TOF Pro (Bruker). For LC-MS/MS injections, samples were separated with the extended Evosep method ''15 samples/day'' keeping the analytical column (PSC-15-100-3-UHPnC, ReproSil c18 3µm 120A 15cm ID 100µm, PepSep) at 50°C. For the dual TIMS TOF, measurements were acquired in diaPASEF mode (data independent acquisition Parallel Accumulation Serial Fragmentation) (9, 10). For the ion mobility settings, the inversed mobilities from 1/K0 0.60 Vs/cm^2 to 1.60 Vs/cm^2 were analyzed with ion accumulation and ramp time of 166 ms, respectively. 1 survey TIMS-MS scan was followed by 16 diaPASEF scans of 25 *m/z* isolation windows (10). Singly charged ions were excluded using the polygon filter mask. For the spectral library, ddaPASEF injections were acquired with the same parameter as above. Except for MS/MS acquisition, isolation windows were set to m/z 2.0 for precursor ions below m/z 700, and m/z 3.0 for precursor ions above.

Protein quantification and proteome Analyses

Data obtained from mass spectrometry was analysed using the program Spectronaut from Biognosys. diaPASEF files were analyzed with the spectral library generated in Pulsar using the default Biognosys parameters for quantifications. For the Pulsar search, raw files were searched against the reviewed Uniprot Human reference database with Trypsin as specific enzyme allowing max. 2 missed cleavages. Acetylation (Protein N-term) and Oxidation (M) were variable modifications and the false discovery rate (FDR) on peptide, Phenol-soluble modulin (PSM) and protein level was set to 1%. A minimum of 2 peptide/protein filter was applied. Only proteins present in 3 replicates or more were considered for the differential expression analysis. Proteins with at least 2-fold change and p-value below 0.05 were chosen as candidates for modulation by FASN inhibition. The list of differentially expressed canidates were tested for over-representation of GO terms in the categories of Biological Process, Cellular Component and Molecular Function using PANTHER (11). The obtained lists of GO terms were then redundancy corrected using REVIGO (12). Differentially expressed proteins were also tested for over-representation of pathways from the Kyoto Encyclopedia of Gene Ontologies (KEEG) (13). STRING Enrichment app was used to identify enriched gene ontology (GO) processes highlighting functional categories represented in proteins DOWN or UP-regulated in organoid derived NSPCs with or without Cerulenin (14). Cytoscape v3.8.2 was used to generate and visualize the obtained protein enrichment maps (15).

Legends Supplemental Tables and Datasets

Table S1. KEEG Pathway over-representation in differentially expressed proteins between Control (EtOH) and Cerulenin treated CD133-sorted NSPCs from human forebrain organoids.

Table S2. Antibodies used in this study.

Dataset S1. All detected proteins between Control (EtOH) and Cerulenin-treated CD133-sorted NSPCs from human forebrain organoids. Four replicates of each condition.

Dataset S2. Differentially expressed proteins between Control (EtOH) and Cerulenin treated CD133-sorted NSPCs from human forebrain organoids.

Dataset S3. Gene ontology consortium over-represented terms for Biological Process in differentially expressed proteins between Control (EtOH) and Cerulenin treated CD133-sorted NSPCs from human forebrain organoids.

Dataset S4. Gene ontology consortium over-represented terms for Cell Component in differentially expressed proteins between Control (EtOH) and Cerulenin treated CD133-sorted NSPCs from human forebrain organoids.

Dataset S5. Gene ontology consortium over-represented terms for Molecular Function in differentially expressed proteins between Control (EtOH) and Cerulenin treated CD133-sorted NSPCs from human forebrain organoids.

Legends Supplemental Movies

Movie S1. Genetic ablation of FASN in forebrain organoids affects polarity of human progenitors.

Forebrain human organoids (30 days in culture) were co-electroporated with NT-gRNA and pMax-GFP and imaged 24h later. 26h time-lapse of one exemplary cortical unit is shown (13 frames, 2h each). Red arrowhead points toward radial glia process of electroporated progenitors. Scale bars represent 50 mm.

Movie S2. Genetic ablation of FASN in forebrain organoids affects polarity of human progenitors.

Forebrain human organoids (30 days in culture) were co-electroporated with *FASN*-gRNA and pMax-GFP and imaged 24h later. 26h time-lapse of one exemplary cortical unit is shown (13 frames, 2h each). Red arrowhead points toward radial glia process of electroporated progenitors. Scale bars represent 50 mm.

Movie S3. Genetic ablation of FASN in forebrain organoids affects polarity of human progenitors.

Three consecutive videos of forebrain human organoids (30 days in culture) were co-electroporated with NT-gRNA and pMax-GFP and imaged 24h later. 26h time-lapse of one exemplary cortical unit is shown (13 frames, 2h each). Scale bars represent 50 mm.

Movie S4. Genetic ablation of FASN in forebrain organoids affects polarity of human progenitors.

Three consecutive videos of forebrain human organoids (30 days in culture) were co-electroporated with *FASN*-gRNA and pMax-GFP and imaged 24h later. 26h time-lapse of one exemplary cortical unit is shown (13 frames, 2h each). Scale bars represent 50 mm.

Supplementary References

1. Gorski JA*, et al.* (2002) Cortical excitatory neurons and glia, but not GABAergic neurons, are produced in the Emx1-expressing lineage. *J Neurosci* 22(15):6309-6314.
2. Chakravarthy MV*, et al.* (2007) Brain fatty acid synthase activates PPARalpha to maintain energy homeostasis. *The Journal of clinical investigation* 117(9):2539-2552.
3. Thomson JA*, et al.* (1998) Embryonic stem cell lines derived from human blastocysts. *Science (New York, N.Y* 282(5391):1145-1147.
4. Bowers M*, et al.* (2020) FASN-Dependent Lipid Metabolism Links Neurogenic Stem/Progenitor Cell Activity to Learning and Memory Deficits. *Cell Stem Cell* 27(1):98-109 e111.
5. Qian X*, et al.* (2016) Brain-Region-Specific Organoids Using Mini-bioreactors for Modeling ZIKV Exposure. *Cell* 165(5):1238-1254.
6. Ran FA*, et al.* (2013) Genome engineering using the CRISPR-Cas9 system. *Nature protocols* 8(11):2281-2308.
7. Jaeger BN*, et al.* (2020) Miniaturization of Smart-seq2 for Single-Cell and Single-Nucleus RNA Sequencing. *STAR Protoc* 1(2):100081.
8. Cardona A*, et al.* (2012) TrakEM2 software for neural circuit reconstruction. *PLoS ONE* 7(6):e38011.
9. Meier F*, et al.* (2018) Online Parallel Accumulation-Serial Fragmentation (PASEF) with a Novel Trapped Ion Mobility Mass Spectrometer. *Mol Cell Proteomics* 17(12):2534-2545.
10. Meier F*, et al.* (2020) diaPASEF: parallel accumulation-serial fragmentation combined with data-independent acquisition. *Nature methods* 17(12):1229-1236.
11. Thomas PD*, et al.* (2006) Applications for protein sequence-function evolution data: mRNA/protein expression analysis and coding SNP scoring tools. *Nucleic Acids Res* 34(Web Server issue):W645-650.
12. Supek F, Bosnjak M, Skunca N, & Smuc T (2011) REVIGO summarizes and visualizes long lists of gene ontology terms. *PLoS ONE* 6(7):e21800.

13. Kanehisa M & Goto S (2000) KEGG: kyoto encyclopedia of genes and genomes. *Nucleic Acids Res* 28(1):27-30.
14. Jensen LJ*, et al.* (2009) STRING 8--a global view on proteins and their functional interactions in 630 organisms. *Nucleic Acids Res* 37(Database issue):D412-416.
15. Shannon P*, et al.* (2003) Cytoscape: a software environment for integrated models of biomolecular interaction networks. *Genome research* 13(11):2498-2504.

Table S1. KEEG Pathway over-representation in differentially expressed proteins between Control (EtOH) and Cerulenin treated CD133-sorted NSPCs from human forebrain organoids.

KEEG Pathways (DOWN)	FDR	matching proteins in your network (labels)
Carbon metabolism	5.21E-08	ALDOC,TPI1,ENO1,PGLS,ACAT2,PGAM1,ALDOA,TKT,GPI,ENO2,MDH1
Glycolysis / Gluconeogenesis	7.09E-08	ALDOC,TPI1,ENO1,PGAM1,PGM2,ALDOA,LDHB,GPI,ENO2
Pentose phosphate pathway	3.25E-06	ALDOC,PGLS,PGM2,ALDOA,TKT,GPI
Biosynthesis of amino acids	1.76E-05	ALDOC,TPI1,ENO1,PGAM1,ALDOA,TKT,ENO2
Metabolic pathways	2.72E-05	GSS,ALDOC,LTA4H,TPI1,ENO1,PGLS,ACLY,PDXK,CMBL,ISYNA1,AK2,FDPS,ACAT2,PGAM1,SEPHS1,PGM2,ALDOA,LDHB,TKT,GPI,IMPA1,GNPDA1,ENO2,MDH1
Regulation of actin cytoskeleton	0.00015	VCL,ACTN4,CRK,PAK2,MSN,ENAH,EZR,TMSB4X,RDX
Proteasome	0.00019	PSMB1,PSMB4,PSMB2,PSME1,PSMA1
Pyruvate metabolism	0.002	ACAT2,GLO1,LDHB,MDH1
Fructose and mannose metabolism	0.0172	ALDOC,TPI1,ALDOA
RNA degradation	0.0178	ENO1,LSM8,LSM3,ENO2
Cysteine and methionine metabolism	0.0303	GSS,LDHB,MDH1
Amino sugar and nucleotide sugar metabolism	0.0351	PGM2,GPI,GNPDA1
Leukocyte transendothelial migration	0.0498	VCL,ACTN4,MSN,EZR
KEEG Pathways (UP)	FDR	matching proteins in your network (labels)
Ferroptosis	0.00012	FTH1,PCBP1,PCBP2,VDAC2,VDAC3
Ascorbate and aldarate metabolism	0.00032	ALDH2,UGDH,ALDH3A2,ALDH7A1
Protein processing in	0.00032	MOGS,LMAN1,SSR4,HS

endoplasmic reticulum		PH1,HSP90AB1,DNAJA1,HYOU1
Ribosome	0.00055	RPL18A,RPS15A,RPL21,RPS26,RPL13A,RPS5
Parkinson's disease	0.00072	UQCRC1,NDUFS7,MT-CO2,ATP5F1,VDAC2,VDAC3
Oxidative phosphorylation	0.003	UQCRC1,NDUFS7,ATP5J2,MT-CO2,ATP5F1
Histidine metabolism	0.003	ALDH2,ALDH3A2,ALDH7A1
Metabolic pathways	0.003	UQCRC1,MOGS,NDUFS7,ALDH2,CAD,GLUD1,ATP5J2,UGDH,IMPDH2,SHMT2,ALDH3A2,MT-CO2,ATP5F1,APRT,ALDH7A1
Huntington's disease	0.003	UQCRC1,NDUFS7,MT-CO2,ATP5F1,VDAC2,VDAC3
beta-Alanine metabolism	0.004	ALDH2,ALDH3A2,ALDH7A1
Necroptosis	0.0051	FTH1,GLUD1,HSP90AB1,VDAC2,VDAC3
Tryptophan metabolism	0.0063	ALDH2,ALDH3A2,ALDH7A1
Pyruvate metabolism	0.0063	ALDH2,ALDH3A2,ALDH7A1
Fatty acid degradation	0.0075	ALDH2,ALDH3A2,ALDH7A1
Viral carcinogenesis	0.0078	CHD4,HNRNPK,CDK1,DDX3X,VDAC3
Valine, leucine and isoleucine degradation	0.0084	ALDH2,ALDH3A2,ALDH7A1
Arginine and proline metabolism	0.0084	ALDH2,ALDH3A2,ALDH7A1
Lysine degradation	0.0131	ALDH2,ALDH3A2,ALDH7A1
Glycerolipid metabolism	0.0131	ALDH2,ALDH3A2,ALDH7A1
Spliceosome	0.0131	RBM25,PCBP1,HNRNPK,DDX39B
Thermogenesis	0.0143	UQCRC1,NDUFS7,ATP5J2,MT-CO2,ATP5F1
Glycolysis / Gluconeogenesis	0.0158	ALDH2,ALDH3A2,ALDH7A1
Alzheimer's disease	0.0265	UQCRC1,NDUFS7,MT-CO2,ATP5F1
Salmonella infection	0.0265	MYH9,MYH10,KLC1

Alanine, aspartate and glutamate metabolism	0.0453	CAD,GLUD1
DNA replication	0.0459	MCM7,RFC5

Table S2. Antibodies used in this study.

Antibodies	Company	Catalogue Number
Mouse anti β-catenin	Millipore	Cat No. 05-665<br>RRID: AB_309887
Rat anti BrdU (1:250)	Abcam	Cat No. ab6326-250<br>RRID: AB_305426
Mouse APC anti human CD133 (1:20)	Biolegend	Cat No. 372806<br>RRID: AB_2632882
Mouse APC isotype control (1:20)	Biolegend	Cat No. 400122<br>RRID: AB_326443
Rabbit anti Cleaved Caspase 3 (1:500)	Cell Signalling Technology	Cat No. 9664<br>RRID: AB_2070042
Rat anti Ctip2 (1:200)	Abcam	Cat No. ab18465<br>RRID: AB_10015215
Rabbit anti FASN (1:200)	Abcam	Cat No. ab22759<br>RRID: AB_732316
Rabbit anti Ki67 (1:500)	Abcam	Cat No. ab16667<br>RRID:AB_302459
Rat anti Ki67 (1:200)	eBioscience	Cat No.14-5698-82<br>RRID:AB_10854564
Mouse anti Nestin (1:200)	Millipore	Cat No. MAB5326<br>RRID: AB_11211837
Mouse anti Nestin (1:200)	BD Biosciences	Cat No. 556309<br>RRID: AB_396354
Goat anti Sox2 (1:200)	Santa Cruz Biotechnology	Cat No. sc17320<br>RRID: AB_2286684
Rabbit anti Tbr1 (1:250)	Abcam	Cat No. ab31940<br>RRID: AB_2200219
Rabbit anti Tbr2 (1:250)	Abcam	Cat No. ab183991<br>RRID: AB_2721040
Rabbit anti Tbr2 (1:200)	Abcam	Cat No. ab23345<br>RRID: AB_778267
Rabbit anti ZO-1 (1:500)	Thermo Fisher Scientific	Cat No. 40-2300<br>RRID: AB_2533457
AffiniPure Donkey Anti-Rat IgG (H+L) coupled to fluorophores	Jackson Immuno Research Labs	Cat No.712-005-150<br>RRID: AB_2340630
AffiniPure Donkey Anti-Rabbit IgG (H+L) coupled to fluorophores	Jackson Immuno Research Labs	Cat No.711-005-152<br>RRID: AB_2340585
AffiniPure Donkey Anti-Goat IgG (H+L) coupled to fluorophores	Jackson Immuno Research Labs	Cat No.705-005-147<br>RRID: AB_2340385
AffiniPure Donkey Anti-MouseIgG (H+L) coupled to fluorophores	Jackson Immuno Research Labs	Cat No.703-005-155<br>RRID: AB_2340346
